# Clinicopathological and Immunohistochemical Profile of Mantle Cell Lymphoma: An Institutional Experience

**DOI:** 10.7759/cureus.16534

**Published:** 2021-07-21

**Authors:** Pritinanda Mishra, Somanath Padhi, Pavithra Ayyanar, Swagatika Samal, Saroj Das Majumdar, Ashutosh Panigrahi, Mukund Sable

**Affiliations:** 1 Pathology, All India Institute of Medical Science (AIIMS), Bhubaneswar, IND; 2 Radiation Oncology, All India Institute of Medical Science (AIIMS), Bhubaneswar, IND; 3 Hematology and Medical Oncology, All India Institute of Medical Science (AIIMS), Bhubaneswar, IND

**Keywords:** mantle cell lymphoma, cyclin d1, sox11, c-myc, tp53, aberrant phenotype, biology

## Abstract

Introduction

Mantle cell lymphoma (MCL) is a biologically aggressive B-cell non-Hodgkin lymphoma (NHL) with distinctive morphologic, immunophenotypic, and molecular characteristics. Differentiation from other chronic lymphoproliferative disorders is essential for prognostication.

Aim

This paper aims to study the clinicopathological features of MCL with emphasis on immunohistochemical features and disease correlation.

Method

To do so, clinicopathological characteristics from 21 cases of MCL (14 males, seven females, M:F=2:1) diagnosed in the last five years i.e. 2015 to 2020, were retrospectively reviewed and correlated with immunohistochemistry (IHC) data. Particularly those pertaining to cyclin D1, SRY-box transcription factor 11 (SOX11), cluster of differentiation (CD) 5, CD23, MIB E3 ubiquitin protein ligase 1 (MIB1), tumor protein 53 (TP53), c-myelocytomatosis oncogene product (c-MYC), multiple myeloma oncogene 1 (MUM1), mouse double minute 2 homolog (MDM2), and Epstein-Barr virus latent membrane protein 1 (EBV-LMP1) expression with its aberrations.

Observations

This study shows that MCL constituted 4.2% (21/500) of all NHLs with a mean age of 57.5 years (median 60 years, range 30 to 80 years). The disease was nodal in 19, and extranodal in the remaining two cases. 14 of 21 (67%) had generalized lymphadenopathy and 71% had bone marrow (BM) involvement. The nodal involvement was diffuse in 9/17 (53%), 8/21 (38%) had a blastoid morphology, and an in-situ MCL pattern was not seen in any of the cases selected for the study. Cyclin D1 immunoexpression correlated well with SOX11; CD5-negative in five cases; and CD23-positive in three cases. TP53 and c-MYC expression were noted in 17/19 (89.4%) and 8/17 (47%), respectively. MUM1 registered positive in six cases. None of the cases showed immunopositivity for MDM2 and EBV-LMP1.

Conclusion

In essence, this study indicates that morphological and immunophenotypic subclassification of mantle cell lymphoma with a wider panel of IHC markers is essential for understanding disease biology and better prognostication.

## Introduction

Mantle cell lymphoma (MCL) is a relatively rare lymphoproliferative neoplasm (LPN), accounting for less than 10% of all non-Hodgkin lymphomas (NHL) [1]. Its morphology is quite homogeneous, but it varies strikingly in about 10% of the cases, making the diagnosis of MCL challenging for histopathologists. The disease is characterized by hallmark translocation t(11; 14) (q13; q32) which juxtaposes with cyclin D1 and the immunoglobulin heavy chain genes resulting in an increased expression of the cyclin D1 protein, which in turn results in cellular proliferation and increased survival [2]. Although morphology and immunohistochemical features [B-lymphocyte antigen cluster of differentiation (CD)20 or CD20-positive(+)/ CD5+/ cyclin D1+/ CD23-negative(-)/ CD10(-)] are characteristic for diagnosis, aberrant phenotypic expressions are not uncommon [3], and these are more commonly reported in blastoid phenotype [4]. Common aberrancies reported in the literature (either in isolation or in combinations) include CD5-, CD23+, CD10+, B-cell lymphoma 6 protein or BCL6+, and cyclin D1- subgroups that may pose a diagnostic dilemma for the surgical pathologist [4-6].

Recently, c-myelocytomatosis oncogene product (c-MYC) and tumor protein 53 (TP53) gene rearrangement were reported to be associated with aggressive biological behavior and overall inferior survival in a subgroup of MCL subjects for which, further in-depth studies are necessary [7,8]. The objective of our present study is to discern the immunophenotypic characteristics of a cohort of MCL cases on lymph node (LN) biopsies and compare them with clinicopathological features and their impact on disease biology. We also present a summary of the relevant published literature pertinent to our observations.

## Materials and methods

The archival biopsy material from the surgical pathology section of the Department of Pathology in a tertiary care center based in eastern India was searched retrospectively for cases of MCL diagnosed over the last five years (2015 to 2020). We included both nodal and extranodal cases in our series, and their morphological and immunohistochemistry (IHC) characteristics were reviewed and analyzed by two independent surgical pathologists (PNM, MNS) as per the criteria proposed by the 2015 World Health Organization (WHO) classification of hematopoietic and lymphoid neoplasm [4]. We included 21/28 MCL cases following stringent criteria where detail morphological and immunophenotypical data of lymph node and bone marrow (BM) biopsy were available for descriptive study. The remaining 7/28 cases in the leukemic phase, diagnosed purely based on peripheral smear and BM morphology with a limited panel of IHC, were excluded as LN biopsies were not performed in those, and flow cytometry immunophenotype data were not available in most of them.

Data pertaining to age, gender, lymphadenopathy, organomegaly, peripheral blood, and BM involvement were collected from medical records. Four microns thick deparaffinized and hematoxylin and eosin (H&E) stained tissue sections were subjected to routine morphological analysis describing the following features: nodal architecture, the morphological phenotype of tumor cell (classical vs. blastoid), angiocentricity, proliferation index [per 10 high power (x400) fields], presence or absence of extranodal spread, high endothelial venules (HEV), pink histiocytes, residual follicles.

The following panel of antibodies was used for further characterization as per protocol with pre-treatment by microwave heating: CD20 (Clone L26, Pathnsitu, 1:100), CD3 (polyclonal, Pathnsitu, 1:100), CD5 [Clone 4C7, Dako, ready to use (RTU)], CD10 (Clone 56Cb, Dako, RTU), CD23 (Clone DAK CD23, Dako, RTU), cyclin D1 (Clone EP12, Dako, RTU), BCL2 (Clone EP36, Pathnsitu, 1:100), BCL6 (polyclonal, Biocare, RTU), TdT (Clone EP299, Pathnsitu, RTU), MIB1 (Dako, 1:150), SOX11 (Clone MRQ58, Cell marque, RTU), TP53 (Clone BP53-12, Pathnsitu, RTU), c-MYC (Clone EP121, Pathnsitu, RTU), MDM2 (Clone SMP14, Sigma-Aldrich, 1:1000), MUM-1 (Clone EP190, Pathnsitu, RTU) and EBV-LMP1 (Clone CS1-4, Dako, RTU).

We followed the cut-off as proposed by Ribera-Cortada et al. for SOX11; (≥10% tumor cells with nuclear expression as positive) [9]. Furthermore, TP53 and c-MYC expression were semi quantified by a histoscore (HScore) calculated by multiplying proportion of positivity [0; negative, 1 (low+); 1-10%+, 2 (intermediate+); 11-50%+, and 3 (high+) (>50%+)] with intensity [1+; weak, 2+; intermediate, 3+; strong]. MDM2 and MUM1 (both nuclear) and EBV-LMP1 (membrane) results were reported as positive or negative. Follow-up data was obtained from telephonic conversations with patients or their next of kin. Overall survival (OS) period was calculated from the date of diagnosis to the date of death or last follow-up.

Statistical analysis

All categorical variables were expressed in frequency and percentage. OS was compared between two subgroups in relation to age (≤ 60 vs. > 60 years), pattern of nodal involvement (diffuse vs. non-diffuse), variant (classical vs. blastoid), MIB1 index (30≤ vs. > 30%), TP53 and c-MYC expression (high/intermediate vs. low/negative), and SOX11 (positive vs. negative). The p-value < 0.05 was considered statistically significant. All statistical analyses were done on IBM SPSS (IBM Corp. Released 2015. IBM SPSS Statistics for Windows, Version 23.0. Armonk, NY: IBM Corp.).

## Results

Clinicopathological features

**Figure 1:**
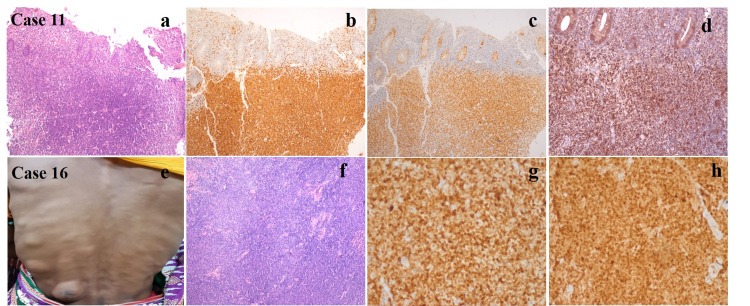
(a) Fragments of colonic mucosa showing infiltration by atypical lymphoid cells H&E 100X, (b) Lymphoid cells are immunopositive for CD5, (c) Cyclin D1, (d) TP53, (e) Skin involvement in MCL presenting as subcutaneous nodules, (f) Classical small cell morphology of MCL. H&E 100X, (g) Lymphoid cells are immunopositive for cyclin D1, (h) SOX11 (IHC X 200X)

The clinicopathological characteristics, immunohistochemical profile of all 21 cases of MCL are presented in the Tables below. These constituted 4.2% of all NHLs (21/500) diagnosed over the last five years at our center. There were 14 males and seven females (M:F=2:1) with a median age at diagnosis of 60 years (range 30 to 81 years). Generalized lymphadenopathy with associated hepatosplenomegaly was noted in 14 (66.7%) and seven (33.3%) of the cases, respectively. Two had extranodal involvement (stomach, caecum, one each). One (case no. 16) had multiple nodular skin lesions (Figure 1) in addition to generalized lymphadenopathy, splenomegaly, and bone marrow involvement. And 15 cases (71.4%) (including positron emission tomography or PET scan finding of one case) had BM involvement at the time of initial diagnosis (Table 1).

**Table 1:** Clinicohematological profile of 21 patients of MCL. Abbreviations: ALC: absolute lymphocyte count, BM: bone marrow, BMBx: bone marrow biopsy, F: female, HSM: hepatosplenomegaly, M: male, LN: lymphadenopathy, LC: lymphoid cell, NA: not available, TLC: total leucocyte count. ≠PETCT showed  F-fluorodeoxyglucose (FDG) uptake in the pelvic bones.

Case no	Age (y)/ Gender	Site	Generalized LN	HSM	Peripheral smear findings			BM aspiration % of atypical lymphoid cells	BMBx	Follow up (months)
					TLC (/cu.mm)	ALC (/cu.mm)	Atypical LC (%)			
1	81/M	Epitrochlear	Present	Splenomegaly	21,000	4200	25	50	Involved	Dead, 2
2	52/M	Cervical	Present	Splenomegaly	15,000	2250	10	22	Involved	Dead, 34
3	54/M	Axillary	Present	HSM	NA	NA	NA	NA	NA	Dead, 10
4	61/F	Epitrochlear	Absent	Absent	9000	2700	00	00	Not involved	Alive, 37
5	49/M	Cervical	Present	Absent	NA	NA	NA	NA	Involved ^≠^	Dead, 21
6	65/F	Cervical	Present	Absent	10390	2078	14	50	Involved	NA
7	66/M	Cervical	Present	Absent	12130	4852	35	30	Involved	Dead, 3
8	61/M	Gastric	Absent	HSM	13000	1560	LEB,12	Dry tap	Involved	Dead, 6
9	71/F	Supraclavicular	Present	Absent	8700	1740	10	15	Involved	Dead, 8
10	60/M	Inguinal	Absent	Absent	7900	1940	00	NA	NA	NA
11	64/M	Caecal growth	Absent	Absent	NA	NA	NA	NA	NA	NA
12	57/F	Axillary	Present	Splenomegaly	8600	4386	10	30	Involved	Alive, 30
13	45/F	Inguinal	NA	NA	NA	NA	NA	NA	NA	NA
14	70/F	Inguinal	Absent	Absent	8720	1744	05	25	Involved	Alive, 46
15	47/M	Cervical	Present	NA	NA	NA	NA	NA	NA	Alive, 18
16	40/F	Cervical, skin lesion	Present	Splenomegaly	10630	2890	05	80	Involved	Dead, 16
17	30/M	Cervical	Present	Splenomegaly	18510	14,580	75	40	Involved	Alive, 10
18	50/M	Supraclavicular	Absent	Absent	52980	43530	35	35	Involved	Dead, 3
19	50/M	Inguinal	Present	Absent	14850	3118	15	10	Involved	Alive, 6
20	60/M	Cervical	Present	Absent	7810	1405	00	05	Involved	Alive, 3
21	74/M	Inguinal	Present	Absent	14000	4620	26	30	Involved	Alive, 2

Histomorphological and immunohistochemical details of cases with extranodal involvement (case no.11 and case no.16) are described in Figure 1.

Histopathological findings

Excision lymph node biopsy material was available in 17 of the cases, needle core LN biopsy in two, and mucosal biopsies in two cases (as above) for histopathological examination (Table 2).

**Table 2:** Histopathological findings in 21 patients of MCL Abbreviations: NA: not available, HEV: high endothelial venules, HPF: high power field, ENE: extranodal extension

	Pattern	In-situ lesion	Pink histiocytes	HEV	Angiocentricity	Residual follicles	Mitosis/10HPF	ENE	Morphological types
1	Diffuse	-	Present	Present	Present	Present	26	Present	Blastoid
2	Diffuse	-	Present	Present	Present	Present	15	Present	Classical
3	Diffuse	-	Present	Present	Present	Absent	14	Absent	Classical
4	Diffuse	-	Present	Present	Present	Present	65	Present	Blastoid
5	Nodular	-	Absent	Absent	Present	Present	15	Present	Classical
6	Diffuse	-	Present	Absent	Present	Present	09	Present	Classical
7	Mixed	-	Present	Present	Present	Absent	78	Present	Blastoid
8	Diffuse	-	Absent	Absent	Present	NA	10	NA	Classical
9	Mixed	-	Absent	Absent	Present	Absent	28	Present	Blastoid
10	Diffuse	-	Present	Absent	Present	Present	10	Present	Classical
11	Diffuse	-	Absent	Absent	Absent	NA	10	NA	Classical
12	Nodular	-	Present	Present	Present	Present	10	Present	Classical
13	Diffuse	-	Present	Present	Present	NA	75	NA	Blastoid
14	Mixed	-	Present	Present	Present	Absent	16	Present	Classical
15	Diffuse	-	Present	Present	Present	Present	10	Present	Classical
16	Mixed	-	Present	Present	Present	Absent	05	Present	Classical
17	Mixed	-	Present	Absent	Absent	Absent	60	Present	Blastoid
18	Diffuse	-	Present	Present	Present	Absent	16	Present	Classical
19	Diffuse	-	Absent	Absent	Absent	NA	50	NA	Blastoid
20	Diffuse	-	Present	Present	Present	Absent	14	Present	Classical
21	Nodular	-	Present	Present	Present	Present	35	Present	Blastoid

Morphologically, the neoplastic lymphoid cells showed a classical small cell morphology with irregular nuclear outline in 13/21 (62%) cases (classical variant); and the remaining eight (38%) cases had a blastoid phenotype with prominent nucleoli and increased mitoses. Pleomorphic variant of MCL was not encountered in any of our cases. Capsular infiltration, tumor angiocentricity; and pink histiocytes were described in 16/17 (94%), 18/21 (85%), and 16/21 (76%) cases, respectively.

Immunohistochemical findings

Classical immunophentype such as CD20-positive/ CD5-positive/ CD10-negative/ CD23-negative/ cyclin D1-positive/ SOX11-positive was seen in 16 cases (76.2%), whereas aberrant phenotype was noted in five (23.8%) (Table 3).

**Table 3:** Immunohistochemical profile of 21 patients of MCL Abbreviations: CD: cluster of differentiation, ND: not done, -: negative, +: positive, +w: weakly positive, Intensity : (+)=weak,  (++)=intermediate, (+++)=strong

	Morphological type of MCL	CD20	CD5	CD23	Cyclin D1	SOX 11	BCL2	CD10	BCL6	TP53	c-Myc	MUM1	MDM2	CD3	EBV-LMP1	MIB-1%
1	Blastoid	+	+	-	+	+	+	-	-	-	5(+)	-	-	-	-	37
2	Classical	+	+	-	+	+	+	-	-	10(+)	-	-	-	-	-	22
3	Classical	+	+	-	+	+	+	-	-	95(+++)	-	-	-	-	-	17
4	Blastoid	+	+	-	+	+	+	-	-	70(++)	70(++)	-	-	-	-	80
5	Classical	+	+	-	+	+	+	-	-	30(+)	-	-	-	-	-	55
6	Classical	+	+	-	+	+	+	-	-	20(++)	85(++)	-	-	-	-	27
7	Blastoid	+	+	-	+	+	+	-	-	90(++)	80(+)	-	-	-	-	90
8	Classical	+	+w	-	+	-	+	-	-	55(++)	-	+	-	-	-	25
9	Blastoid	+	+	-	+	+	+	-	-	100(+++)	70(+)	-	-	-	-	80
10	Classical	+	+	-	+	+	+	-	-	-	20(+)	-	-	-	-	25
11	Classical	+	+	-	+	+	+	-	-	100(+++)	ND	-	-	-	-	10
12	Classical	+	+	-	+	+	+	-	-	30(+)	10(+)	-	-	-	-	18
13	Blastoid	+	-	-	+	-	+	-	-	40(++)	ND	+	-	-	-	80
14	Classical	+	-	-	+	+	+	-	-	80(++)	-	+w	-	-	-	40
15	Classical	+	+	-	+	+	ND	-	ND	ND	ND	ND	ND	-	-	10
16	Classical	+	+	-	+	+	+	-	-	80(++)	-	-	-	-	-	35
17	Blastoid	+	+w	-	+	-	+	-	-	90(+++)	-	+w	-	-	-	70
18	Classical	+	+	+	+	ND	ND	-	ND	ND	ND	ND	ND	-	-	40
19	Blastoid	+	+	+w	+	+	+	-	+w	95(+++)	80(+++)	-	-	-	-	68
20	Classical	+	+	+w	+	+	+	-	-	100(++)	-	+	-	-	-	50
21	Blastoid	+	+w	-	+	+	+	-	-	70(+)	-	+	-	-	-	35

**Figure 2:**
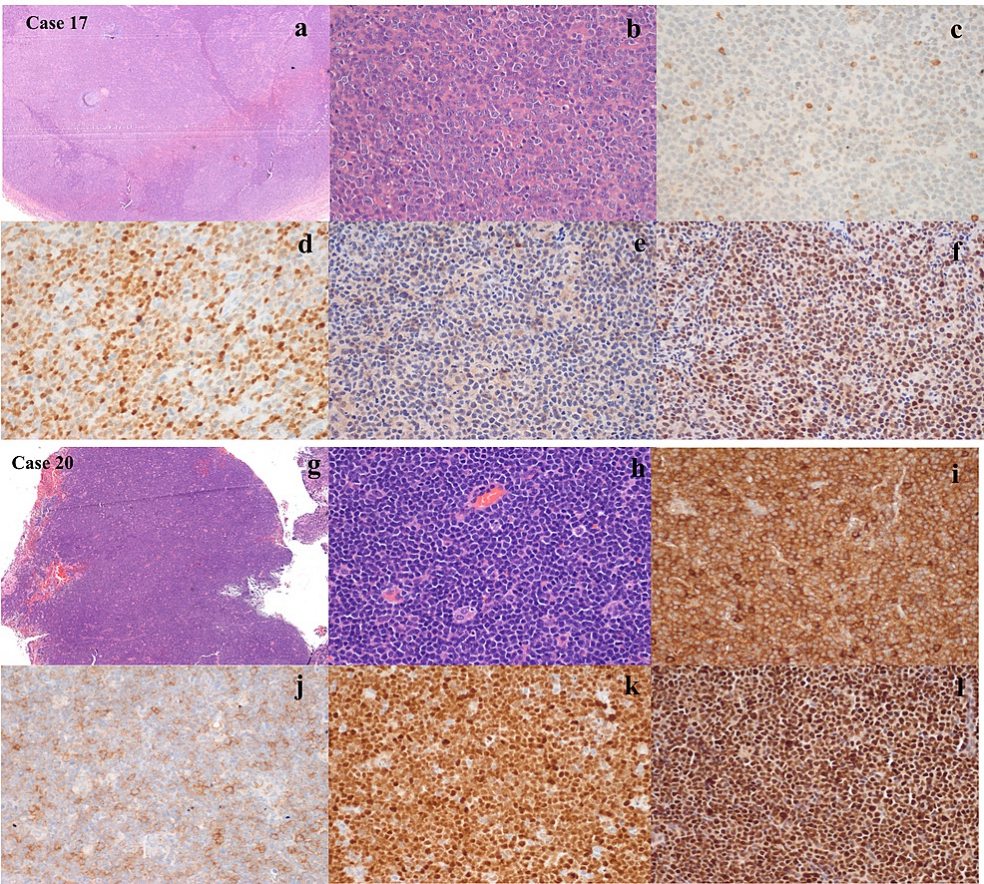
(a) Nodular effacement of the lymph node, H&E 40X, (b) Atypical lymphoid cells are large with prominent nucleoli, blastoid morphology. H&E 200X, (c) Lymphoid cells are immunonegative for CD5, (d) Immunopositive for cyclin D1, (e) Immunonegative for SOX11, (f) Immunopositive for TP53. (IHC X200), (g) Diffuse infiltration of atypical lymphoid cells. H&E 40X, (h) Prominence of pink histiocytes. H&E 100X, (i) Lymphoid cells are immunopositive for CD5, (j) Immunopositive for CD23, (k) Immunopositive for cyclin D1, (l) Immunopositive for SOX11. IHC X 200

Common aberrancies observed were as follows: CD5 (negative to weak+); n=5 (three blastoid, two classical), SOX11-negative; n=3 (two blastoids), CD23+/weak+; n=3 (two classical, one blastoid), BCl6weak+; n=1 (blastoid). While cyclin D1 expression was noted in all cases (21/21, 100%), TP53 expression was noted in 17/21 (80.9%) cases (seven with blastoid phenotype, high/strong in five, intermediate in eight, weak in four); and MUM1-positive was noted in 6/19 cases (31.5%). Similarly, c-MYC expression was noted in 8/17 (47%) cases [high to intermediate in three (two blastoids), and weak in five]. The mean MIB1 proliferation rate of classical and blastoid subgroups were 28.8% and 67.5%, respectively. MDM2 and EBV-LMP1 were negative in all cases. Histomorphological and immunohistochemical details of aberrant cases (case no.17 and case no.20) are described in Figure 2.

None of the cases showed immunoprofile as CD5-negative/CD23-positive/SOX11-negative.

Hematological evaluation

Complete blood counts and BM data were available in 16 cases and 15 cases, respectively. Circulating neoplastic lymphoid cells were evident in 13/16 (81%) which ranged from 5% to 75% of differential leukocyte count (median=14%). 6/8 (75%) blastoid MCL had peripheral blood spillage compared to 7/13 (53.8%) classical variant. BM involvement was reported in 14/15 (93.3%) cases with the lymphoma cells comprising of 5% to 80% of marrow nucleated cells (median=15) in aspirate smears (case no.5 showed F-fluorodeoxyglucose (FDG) uptake in the pelvic bones, and bone marrow examination was not available). The pattern of BM involvement in Table 1 was reported as follows: nodular (n=5), paratrabecular (n=2), mixed (n=5), diffuse (n=2).

Follow-up and overall survival in various prognostic subgroups

The median duration of follow-up post-therapy (n=17/21) was 10 months (range two to 46 months); with nine (52.9%) dead at the time of the last follow-up. The overall survival did not vary between two subgroups in regard to age (60≤ vs. >60 years, 29.6 vs. 22.3 months, respectively, log-rank P=0.067), morphology (classic vs. blastoid, 23.72 vs. 19.54 months, respectively, log-rank P=0.769), TP53 expression (high vs. weak-intermediate, 22.23 vs. 29.66 months, respectively, log-rank P=0.604), c-MYC expression (positive vs. negative, 19.05 vs. 23.1 months, respectively, log-rank P=0.881), SOX11 (positive vs. negative, 25 vs. 8.0months, respectively, log-rank P=0.636); and proliferation rate (low vs. high, 22.3 vs. 23.0 months, respectively, log-rank P=0.961).

## Discussion

We studied 21 cases of MCL diagnosed over a period of five years that occurred in the elderly age group with advanced disease at presentation. The prevalence in our series (4.2%) was slightly more than two other Indian series where it was reported to be 2.1% and 3.4%, respectively [1,10]. Compared to a large series by Hrgovic et al. where 22 cases of cutaneous involvement by MCL was reported, we found such occurrence in one case only and the other two had focal gastrointestinal involvement [4,11]. Classical morphology dominated the blastoid phenotype (13 vs. eight, respectively). Immunophenotypic aberrancies such as CD5-negative, CD23-positive, SOX11-negative were noted (9.5%, 14.3%, and 14.3% cases respectively), and TP53 and c-MYC expression were observed in a good number/higher proportion of our cases. However, none of the clinicopathological characteristics impacted the overall survival.

The pattern of nodal histomorphology in MCL varies from diffuse to nodular-diffuse or a mantle cell pattern. From a morphological point of view, the greatest challenge is to differentiate the latter from its precursor state, the so-called ‘In-situ mantle cell neoplasia (ISMCN)’. ISMCN is characterized by preserved nodal architecture with reactive follicles without an expanded mantle, which characteristically harbors cyclin D1-positive cells confined to the inner zone in some of the follicles. In contrast, the MCL with a mantle cell pattern could be recognized by its more numerous and crowded follicles with a decreased interfollicular area and expanded mantle zone, and the zone showing diffuse nuclear positivity for cyclin D1. The mantle cell pattern of MCL was not seen in our series. Though the 'in-situ MCL is an incidental finding, its pathological significance remains that these may represent the precursor stage of lymphomagenesis with similar molecular signature and confer a favorable prognosis [12]. We did not find any evidence of ‘in-situ lesion’ in our cases; a feature pointing towards an advanced disease with an increased propensity for peripheral blood spillage.

The blastoid variant exhibits large cells with nucleoli and a very brisk mitotic activity (>20-30/10 HPF) and sometimes may mimic a lymphoblastic lymphoma. Cyclin D1 positivity and absence of terminal deoxynucleotidyl transferase (TdT) helps in differentiating the two. The nodular pattern of involvement of the nodes allows other low-grade lymphomas like chronic lymphocytic leukemia (CLL) or follicular lymphoma (FL) into the differentials. The absence of nuclear indentations, presence of pro-lymphocytes, and proliferation centers negate MCL. A mixture of cleaved and non-cleaved cells in a follicular pattern favor FL. In addition, pink histiocytes, high endothelial venules, and angiocentric patterns of atypical lymphoid cells are subtle clues for the diagnosis of MCL [13]. Though they are morphological clues for aiding the diagnosis, their prognostic significance is questionable.

Immunophenotypic aberrations in MCL have been described in the literature [3,5,6]. This possibly results due to origin from the germinal center and/or post germinal center B lymphocytes through the somatic hypermutation of the immunoglobulin heavy-chain variable region (IgVH), especially IGHV3-21, IGHV3-23, IGHV4-34, and IGHV4-59 [14].

Classical immunophenotype of MCL is CD20-positive/ CD5-positive/ cyclin D1-positive/ CD23-negative/ CD10-negative. Though cyclin D1 is one of the most persistent nuclear markers for MCL, it can be absent in a subset of lymphoma known as cyclin D1-negative MCL. This subgroup was clinically aggressive with advanced stage at diagnosis with frequent peripheral blood and extranodal involvement; thus a poor response to therapy [15]. Hence, it is important to diagnose this entity, so that other treatment options could be offered in these groups of MCL. In our series, cyclin D1 was universally expressed by MCL cells; though blastoid phenotype had heterogeneous nuclear positivity compared to stronger intensity among classical subgroup [16].

The largest series by Miao et al. has shown 6% absence of CD5 expression [17] and in the present study, CD5 was not seen in 9% of cases (2/21) [Blastoid (1), Classical (1)], though weak expression was observed in 14% of cases (3/21). Shih et al. showed that all CD5-negative MCL have a classical morphology [18]. A plausible explanation for these differences could possibly be attributed to the lesser number of cases included in the study groups. CD23 is frequently negative in MCL. A study by Saksena et al. has shown that 13% of MCL cases showed CD23 expression and these cases are associated with leucocytosis, a leukemic presentation, bone marrow involvement, CD200 expression, and a lower frequency of SOX11 positivity [19]. In the present study, 14% (3/21) of cases showed membrane positivity for CD23 (one strong expression and two weak expressions) and 66.6% of patients had an elevated leukocyte count and all of them showed bone marrow involvement.

SOX11 immuno-expression was absent in three of our cases (15%) (two blastoid phenotypes). Xu et al. described a large series (n=75) of SOX11-negative MCL and reported that such subgroups have classic morphology, increased CD23 coexpression, lower proliferation rate, increased extranodal involvement, and more propensity for leukemic transformation compared to the SOX11-positive subgroup [20]. Similar observations were also made by Nygren et al. who found that the SOX11-negative group has significantly lower OS compared with SOX11-positive cases (median OS: 1.5 vs. 3.2 years, respectively; P=0.014) [21]. In contrast, the SOX11-negative phenotype offered a significantly better survival advantage over the positive subgroup (five-year OS: 78% vs. 38%, respectively, P=0.001) in another study by Fernandez et al. [22].

In previous studies, the percentages of MUM1 immunoexpression in MCL ranged from 11% to 36% [23,24]. The present study showed positivity in 28% of cases. Though MDM2 expression is associated with poor prognosis in MCL, our study shows that all cases of MCL were immunonegative for MDM2 [25]. Similarly, very few reports showed immunopositivity for EBV-LMP1 [26].

Strong immunoexpression of TP53 and c-MYC in MCL are usually seen with blastoid and pleomorphic morphology and are associated with aggressive behavior and poor outcome. None of the classical cases showed a strong intensity of c-MYC or TP53 staining [16]. However, in contrast to previous observations, the present study reveals the strong immunoexpression of TP53 in two cases of classical MCL and intermediate immunoexpression in five cases of classical MCL.

## Conclusions

We studied the clinicopathological and immunohistochemical features of MCL in our population. Also, the aberrant phenotypic alterations and their clinicopathological correlation were noted. Histomorphology augmented by routine IHC and prognostic markers will help inform the disease status at the time of diagnosis and give clues on further management and close follow-up.
